# MiR-361-3p regulates ERK1/2-induced EMT via DUSP2 mRNA degradation in pancreatic ductal adenocarcinoma

**DOI:** 10.1038/s41419-018-0839-8

**Published:** 2018-07-24

**Authors:** Jisheng Hu, Le Li, Hongze Chen, Guangquan Zhang, Huan Liu, Rui Kong, Hua Chen, Yongwei Wang, Yilong Li, Fengyu Tian, Xinjian Lv, Guanqun Li, Bei Sun

**Affiliations:** Department of Pancreatic and Biliary Surgery, The First Affiliated Hospital of Harbin Medical University, 23 Youzheng Street, Nangang District, Harbin, 150001 Heilongjiang Province China

## Abstract

Metastasis remains one of the most intractable challenges in pancreatic ductal adenocarcinoma (PDAC) biology, and epithelial-to-mesenchymal transition (EMT) is essential to the epithelium-originated solid tumor metastasis cascade. Emerging evidence demonstrates that aberrant miRNA expression is involved in pancreatic cancer progression. We found that miR-361-3p was associated with an advanced stage of PDAC and poor prognosis. Hence, the effect of miR-361-3p on metastasis of PDAC cells was evaluated using Transwell assay and wound healing assay in vitro as well as orthotopic and liver metastasis pancreatic cancer models in vivo. Overexpression of miR-361-3p promoted pancreatic cancer cell migration and invasion in vitro, and miR-361-3p-elevated PDAC cells were prone to generating metastatic nodules in vivo. However, miR-361-3p showed no significant effect on the proliferation of PDAC cells in vivo or in vitro. Further study demonstrated that miR-361-3p could enhance EMT and ERK pathway activation, and ERK inhibitor could attenuate miR-361-3p-induced EMT. Luciferase assays, qPCR, and western blot and Ago2 co-immunoprecipitation were performed to identify the direct target of miR-361-3p. Mechanistic investigations identified DUSP2 as a direct target of miR-361-3p, and DUSP2 was revealed to be involved in miR-361-3p-induced EMT by directly leading to the inactivation of the ERK pathway. Moreover, we found that miR-361-3p-induced EMT was dependent on Ago2, the core component of RNA-induced silencing complex, while enforced expression of Ago2 enhanced the miR-361-3p-induced effect by promoting interference efficacy and specificity rather than regulating miR-361-3p stability and biogenesis. Thus, this study revealed that miR-361-3p functions as an oncomiR for promoting metastasis and identified the miR-361-3p/DUSP2/ERK axis as a novel EMT axis dependent on Ago2 in PDAC.

## Background

Despite tremendous effort and huge investment to improve the diagnosis and treatment of pancreatic ductal adenocarcinoma (PDAC), PDAC remains one of most lethal malignant tumors, with only an 8% 5-year overall survival rate^1^. The late stage, at which most patients are diagnosed, disqualifies 80% of cases as candidates for radical operation^1^; even after curative resection, most patients will eventually die of early metastases and recurrences. Thus, there is an urgent need for a potential strategy to track and prevent metastatic tumors. The process of metastasis establishes a more invasive and motile cell phenotype in order to escape the primary site and spread into the surrounding or distant tissues. Epithelial-to-mesenchymal transition (EMT) is a cellular process that features loss of epithelial characteristics and acquisition of mesenchymal phenotypes that drive epithelial cells to acquire certain traits, most notably motility and invasiveness^2,3^. Despite evidence that appears to indicate differently^4^, we continue to consider the EMT progress as a crucial role function in pancreatic cancer metastasis^5,6^.

In addition to some core transcription factors regulating EMT, such as Snail1, Snail2, Zeb1, Zeb2, Twist1, and Twist2, all major developmental signaling pathways have also been implicated in some aspect of the EMT program^7,8^; among these, the transforming growth factor-β (TGF-β) signaling pathway has a predominant role^9^. Emerging evidence suggests that extracellular signal-regulated kinases (ERK) are involved in TGF-β-mediated EMT^10–12^. Moreover, ERK pathway activation triggered by RAS or RAF also contributes to EMT^13,14^, and ERK1/2 blockade restrains EMT in lung cancer cells^15^. These findings indicate that the ERK pathway may function as an EMT inducer.

MicroRNAs (miRNAs) are a family of endogenous small non-coding RNAs (ncRNAs) of 19–25 nt that negatively regulate gene expression in a sequence-specific manner at the post-transcriptional level^16^. MiRNAs mediate gene silencing mainly by binding to the 3′-untranslated region (3′-UTR) of target mRNA, resulting in mRNA cleavage^17^ or translational repression^18^. This miRNA-mediated regulation, proved by numerous studies, plays critical roles in pancreatic cancer development and progression^19^. Several miRNAs, including miR-10a^20^, miR-10b^21^, miR-21^22^, and miR-92b-3p^23^, have been characterized for their role in regulating PDAC invasion and metastasis. MiRNAs can mediate tumor metastasis through multiple mechanisms, such as EMT, cancer stem cells (CSC), matrix metalloproteinases (MMPs) activity and some signaling pathways.

Functioning as a guide to specifically recognize target mRNAs, miRNA could be loaded onto Argonaute (Ago) to form an RNA-induced silencing complex (RISC) during assembly, as the Ago protein family is thought to be a core component of the silencing complex^24^. Among the human Ago family (Ago1-4), Ago2 is the only mammalian Ago protein with intrinsic endonuclease activity that, can act as a “Slicer”^25^ in mediating mRNA cleavage. In addition to regulation of RNA-interference (RNAi), Ago2 also mediates endogenous miRNA biogenesis in multiple ways^26^. Hence, the specific biological function of Ago2 on a specific miRNA should be further discussed.

In this study, we discovered that miR-361-3p functions as a novel promoter in tumor metastasis and the EMT process in pancreatic cancer. Moreover, we identified miR-361-3p as a direct negative regulator of dual-specificity phosphatase–2 (DUSP2) via mRNA degradation, thereby activating ERK signaling. Importantly, the role of Ago2 was further demonstrated in miR-361-3p-mediated DUSP2 mRNA cleavage, and high levels of a combination of miR-361-3p and AGO2 were associated with poor prognosis.

## Results

### MiR-361-3p drives pancreatic cancer cell metastasis and is correlated with shorter survival in PDAC

**Fig. 1:**
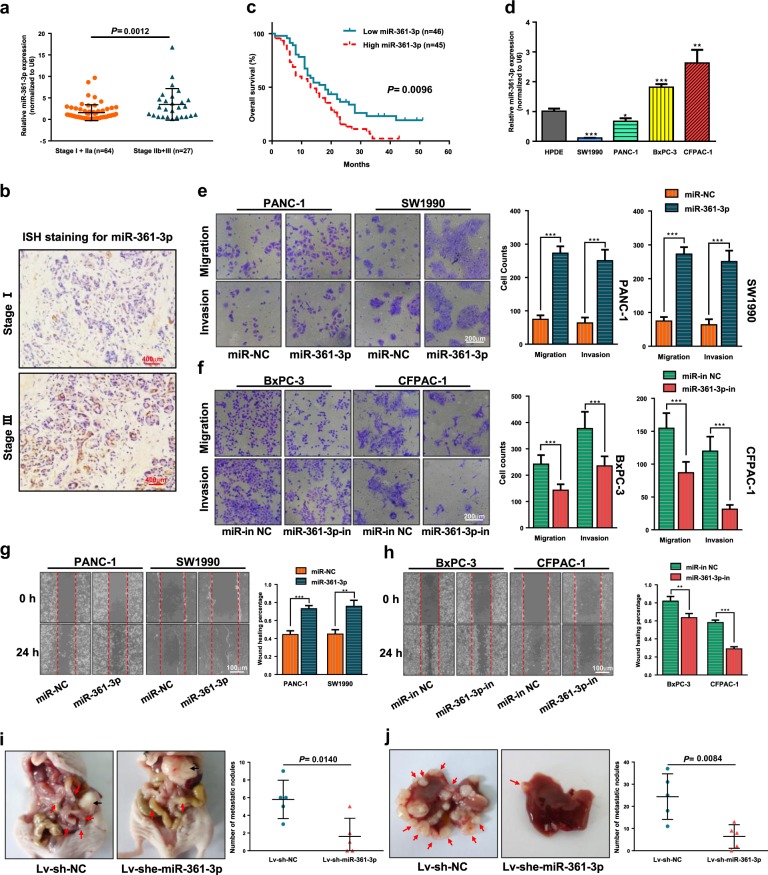
MiR-361-3p overexpression in PDAC correlates with metastasis and shorter survival. **a** Association analyses of miR-361b-3p expression levels and TNM stage. **b** Representative images of the ISH staining analyses of different stage PDAC tissues using anti-miR-361-3p probe. **c** Association between postoperative overall survival (OS) and tumor miR-361b-3p expression levels in 91 PDAC tissues. The low and high levels of miR-361-3p expression are separated according to the median value. **d** Comparison of miR-361-3p expression levels in Bxpc-3, Panc-1, CFPAC, SW1990, and HPDE cell lines. **e-h** Transwell migration/invasion assay and wound healing assay were performed to test the effect of miR-361-3p on PDAC cells metastasis in vitro. **i** Representative images of orthotopic xenograft pancreatic cancer mouse models from two groups (left panel) and metastatic nodes were calculated (right panel), black arrow indicates primary tumor and red arrows indicate metastatic lesions. **j** Representative images of liver metastasis model (left panel) and visible metastatic nodes were calculated (right panel), red arrows indicate metastatic lesions. The statistical significance between different groups was calculated with Student *t* test. Data are shown as the mean ± SD of three replicates; **P < 0.01; ***P < 0.001; ns not significant

QRT-PCR results showed that higher miR-361-3p expression was associated with an advanced cancer stage (Fig. 1a). In situ hybridization (ISH) staining confirmed significantly higher miR-361-3p expression in advanced stage PDAC compared to that of the control and identified the location of miR-361-3p in PDAC tissues (Fig. 1b). Moreover, patients whose tumor expressed a high level of miR-361-3p had a significantly shorter survival time than those whose tumor expressed a low level of miR-361-3p (Fig. 1c). The prognosis of I/IIa and IIb/III patients were separately analyzed and illustrated in Fig. S1a.

To assess the effect of miR-361-3p on the potency of migration and invasion of pancreatic cancer cells, four pancreatic cancer cell lines (BxPC-3, PANC-1, CFPAC-1, and SW1990) were introduced; the relative levels of miR-361-3p in four cancer cell lines and HPDE are illustrated in Fig. 1d, and the transfection efficiency is shown in Fig. S1b–d. Transwell and wound healing assays demonstrated that enhanced expression of miR-361-3p led to a significant increase in the migratory and invasive capability of PANC-1 and SW1990 cells (Fig. 1e, g). Correspondingly, miR-361-3p knockdown led to significantly inhibited cell migration and invasion in BxPC-3 and CFPAC-1 cells (Fig. 1f, h, Fig. S1e).

Moreover, an orthotopic and metastatic pancreatic tumor model with stable transfected BxPC-3-Luc cells lines (Lv-sh-NC and Lv-sh-miR-361-3p) was constructed to determine whether miR-361-3p promotes pancreatic cell metastasis in vivo and miR-361-3p levels of stable cell lines were demonstrated in Fig. S1f. In the orthotopic tumor model, fewer visible metastasis nodes were found in the gut, mesentery, liver, kidney and spleen, compared with the negative group (Fig. 1i), and consistent result was observed in liver metastasis models (Fig. 1j). Such in vivo results were verified again by intravenous injection of antagomir into the orthotopic tumor model (Fig S1i). However, neither small animal imaging nor orthotopic tumor size after sacrifice showed a significant difference in proliferation between the two groups (Fig. S2a-c, f); the in vitro proliferation, EdU and Colony formation assays returned similar results (Fig. S2d-e). MiR-361-3p levels of tissues from the in vivo experiments are illustrated in Fig. S1g-h. Together, these results indicate that miR-361-3p may induce pancreatic cancer cell metastasis and predict a poor prognosis.

### MiR-361-3p promotes EMT via ERK1/2 activation in pancreatic cancer cells

**Fig. 2:**
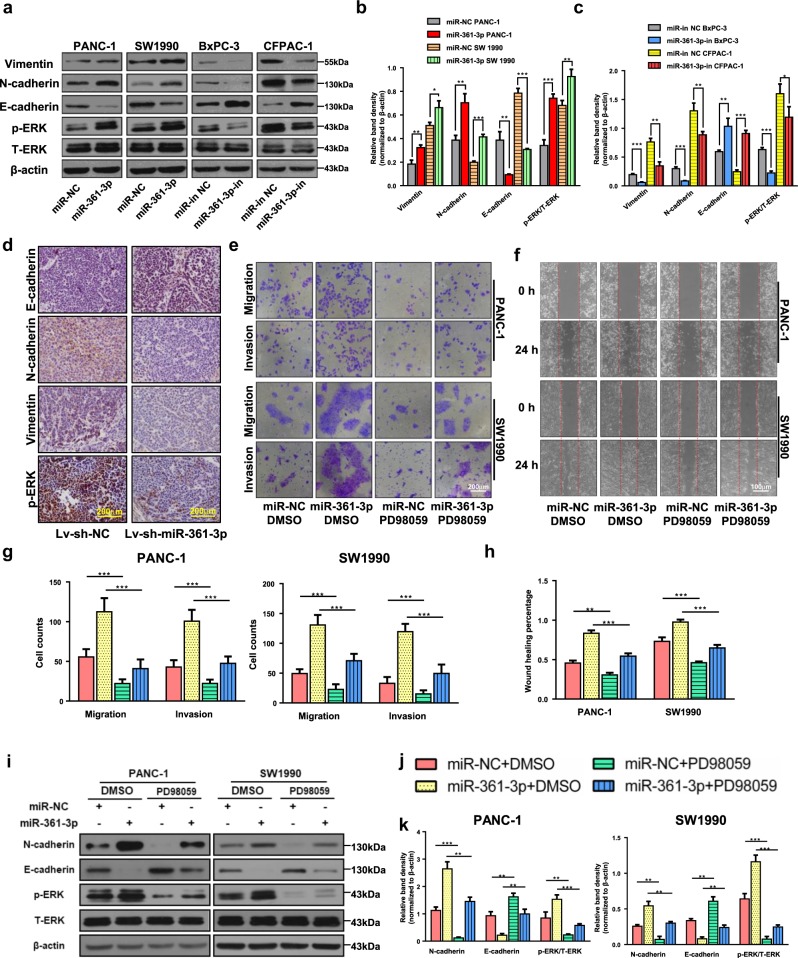
MiR-361-3p induces EMT by ERK1/2 activation. **a**–**c** The expression of Vimentin, N-cadherin, E-cadherin, phospho-ERK1/2, and total ERK1/2 was determined by western blot assays in four pancreatic cancer cell lines transiently transfected with miR-361-3p mimic or miR-361-3p inhibitor. **d** The expression of Vimentin, N-cadherin, E-cadherin, and phospho-ERK1/2 were analyzed in orthotopic tumors by Immunohistochemistry. **e**–**h, j** The Transwell assay and wound healing assay of PANC-1 and SW1990 in negative control, miR-361-3p mimic, PD98059, and miR-361-3p mimic plus PD98059. **i**–**k** Whole PANC-1 and SW1990 lysates from four groups above were subjected to western blot to test N-cadherin, E-cadherin and phospho-ERK1/2. Data are shown as the mean ± SD of three replicates; *P < 0.05; **P < 0.01; ***P < 0.001

EMT enables tumor cells to acquire the features of high motility and invasiveness. To investigate how miR-361-3p regulates pancreatic cancer cell migration and invasion, epithelial and mesenchymal markers were examined. Western blot results showed that miR-361-3p overexpression in PANC-1 and SW1990 cells caused a significant increase in the expression of mesenchymal markers (N-cadherin, Vimentin) and a decrease in an epithelial marker (E-cadherin) (Fig. 2a, b), while miR-361-3p knockdown attenuated EMT in vitro and in vivo (Fig. 2a–d and Fig. S3a-b). Since EMT may have a role in drug resistance^4^, we also interrogated the role of miR-361-3p in gemcitabine resistance. CCK-8 assays and IC50 values demonstrated that miR-361-3p enhanced resistance to gemcitabine treatment (Fig. S3d-e).

The findings of an association between miR-361-3p and EMT prompted us to explore the molecular mechanisms regulated by miR-361-3p. The requirement for ERK1/2 signaling pathway activity in EMT has been demonstrated in several studies^10,13,27^. We researched the potential involvement and effects of ERK1/2 activation in miR-361-3p-induced EMT. First, the effects of miR-361-3p expression on ERK1/2 phosphorylation were determined by western blot in pancreatic cancer cells PANC-1 and SW1990. ERK1/2 phosphorylation was significantly enhanced by miR-361-3p overexpression but decreased when transfected with miR-361-3p knockdown (Fig. 2a–d and Fig. S3a-b). Then, we found that the blockade of ERK1/2 activity by treatment for 48 h with 25 μM PD 98059, a specific inhibitor of MAPKK, alleviates the effects of miR-361-3p on cell migration, invasion and EMT in PANC-1 and SW1990 (Fig. 2e–k). Therefore, the data above suggest that miR-361-3p promotes EMT by activating ERK1/2 in pancreatic cancer cells.

### MiR-361-3p directly targets DUSP2 in pancreatic cancer

**Fig. 3:**
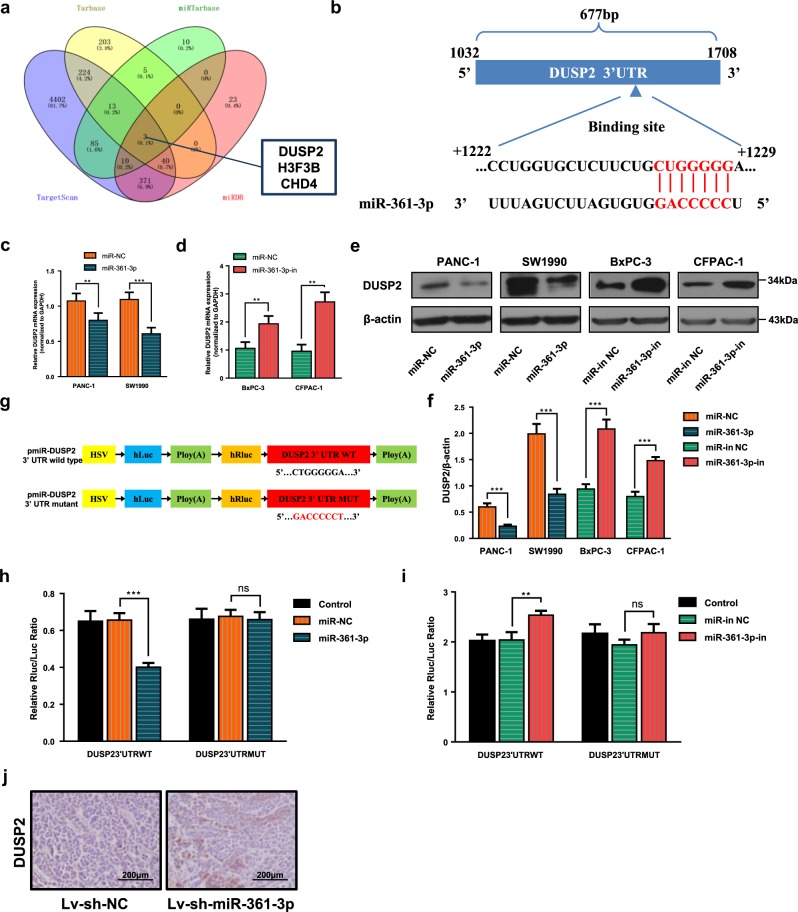
DUSP2 is a target of miR-361-3p. **a** Intersections among four miRNA target prediction algorithms. **b** Schematic representation of DUSP2 3′ UTR indicating the predicted binding site of miR-361-3p. **c**–**f** Relative expression of DUSP2 mRNA and protein after treatment with miR-361-3p mimic or inhibitor. **g** Schematic diagram of the luciferase reporter plasmids of pMIR-wild type-DUSP2 3′ UTR and pMIR-mutant-DUSP2 3′ UTR. **h**, **i** Luciferase activity assays was performed to confirm the direct binding efficiency of miR-361-3p and its putative target DUSP2. **j** Representative images of immunohistochemistry analyses to detect the DUSP2 protein in orthotopic tumors from negative control and miR-361-3p knockdown group. Data are shown as the mean ± SD of three replicates; *P < 0.05; **P < 0.01; ***P < 0.001; ns not significant

To identify the putative target of miR-361-3p, online miRNA target analysis algorithms (TargetScan release 7.1^28^ and miRDB^29^) and validated targets supported by miRTarbase Release 7.0^30^ and Tarbase v7.0^31^ were used simultaneously. DUSP2, H3F3B and CHD4 were found at the intersection of the four databases (Fig. 3a). We focused on DUSP2 because of its negative regulation of ERK1/2 by dephosphorylating both Thr and Tyr residues^32,33^. Thus, we speculated that DUSP2 might be a potential target of miR-361-3p, and predicted binding site is shown in Fig. 3b. To validate this potential regulation, we overexpressed and knocked down miR-361-3p with mimic or miRNA-inhibitor in pancreatic cancer cells. Real-time PCR showed that the miR-361-3p mimic significantly reduced the DUSP2 mRNA level and that the miR-361-3p inhibitor remarkably elevated the DUSP2 mRNA level compared to that in their respective negative controls (Fig. 3c, d). Consistently, the DUSP2 protein level was significantly reduced in miR-361-3p mimic-transfected pancreatic cancer cells but was increased in miR-361-3p-knockdown cells (Fig. 3e, f, j and Fig. S5a-b). Furthermore, to confirm the direct interaction between miR-361-3p and DUSP2 mRNA 3′ UTR, we cloned the full-length wild-type and mutated same length seed sequences of the 3′ UTR of DUSP2 mRNA into pmiR-RB-Report^TM^ vector to conduct a Dual-Luciferase Reporter gene assay (Fig. 3g). The results demonstrated that overexpression of miR-361-3p significantly repressed the relative Luciferase activity, whereas the miR-361-3p inhibitor significantly enhanced it, compared to that in the control group and corresponding negative control. However, such effects were abrogated when the predicted binding site was mutated (Fig. 3h, i). These results indicated that miR-361-3p directly suppressed DUSP2 expression by binding to its 3′ UTR and leading to the degradation of its mRNA.

### DUSP2 was involved in miR-361-3p-mediated ERK1/2 activation and the EMT process

**Fig. 4:**
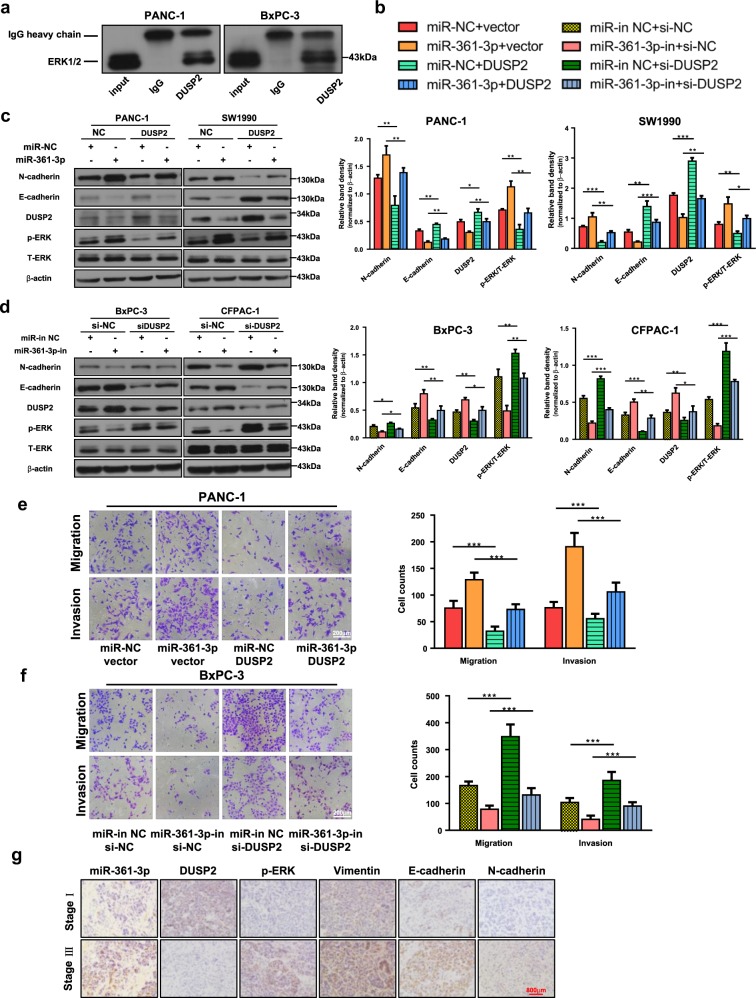
DUSP2 is involved in miR-361-3p-induced EMT and ERK activation. **a** DUSP2 was immunoprecipitated from cell lysates of BxPC-3 and PANC-1 and ERK1/2 was detected using ERK1/2 antibody. **b**, **c** PANC-1 and SW1990 cells were transfected with miR-361-3p mimic, DUSP2 plasmid alone, or the combination of miR-361-3p mimic and DUSP2 plasmid. Forty-eight hours after transfection, the cell lysates were subjected to western blot. **b**, **d** BxPC-3 and CFPAC-1 were transfected with miR-361-3p inhibitor, si-DUSP2 alone, or the combination of miR-361-3p inhibitor and si-DUSP2. 48 h after transfection, the cell lysates were subjected to western blot. **b**, **e**, **f** The role of DUSP2 in miR-361-3p-induced migration and invasion was demonstrated by Transwell assay in PANC-1 and BxPC-3 cell lines. **g** Clinical specimens of PDAC with low and high TNM stages were stained for miR-361-3p, DUSP2, p-ERK1/2, Vimentin, E-cadherin, and N-cadherin. Representative images from a tissue microarray are shown. Data are shown as the mean ± SD of three replicates; *P < 0.05; **P < 0.01; ***P < 0.001

To ascertain if DUSP2 interacts with ERK1/2 in pancreatic cancer cells, DUSP2 was immunoprecipitated from cell lysates of BxPC-3 and PANC-1. As shown in Fig. 4a, the specific interaction between DUSP2 and ERK1/2 was confirmed by a co-immunoprecipitation (co-IP) assay using anti-ERK1/2 antibody. Then, to verify the role of DUSP2 in miR-361-3p-induced ERK1/2 activation and EMT in vitro, siRNA against DUSP2 and DUSP2 overexpression plasmid were introduced. DUSP2 overexpression partly abolished the increase in miR-361-3p-induced phospho-ERK1/2 (p-ERK1/2) levels, N-cadherin levels (Fig. 4c), cell migration and invasion (Fig. 4e). Consistently, DUSP2 knockdown reversed ERK1/2 inactivation, epithelial phenotype (Fig. 4d), inhibition of migration and invasion (Fig. 4f) due to miR-361-3p-inhibitor. Moreover, DUSP2, p-ERK1/2 and EMT-related markers were examined in human PDAC tissues, which revealed that the miR-361-3p level in cancer tissues correlated negatively with the expression of DUSP2 and E-cadherin and positively with p-ERK, Vimentin and N-cadherin, respectively. Together, these data indicated that DUSP2 restoration in pancreatic cancer cell could reverse mesenchymal transition and ERK1/2 pathway activation caused by miR-361-3p, and the miR-61-3p/DUSP2/ERK axis in programming pancreatic cancer EMT was further demonstrated by in vitro experiments and clinical samples.

### Argonaute 2 enhanced miR-361-3p-mediated EMT, ERK1/2 activation

**Fig. 5:**
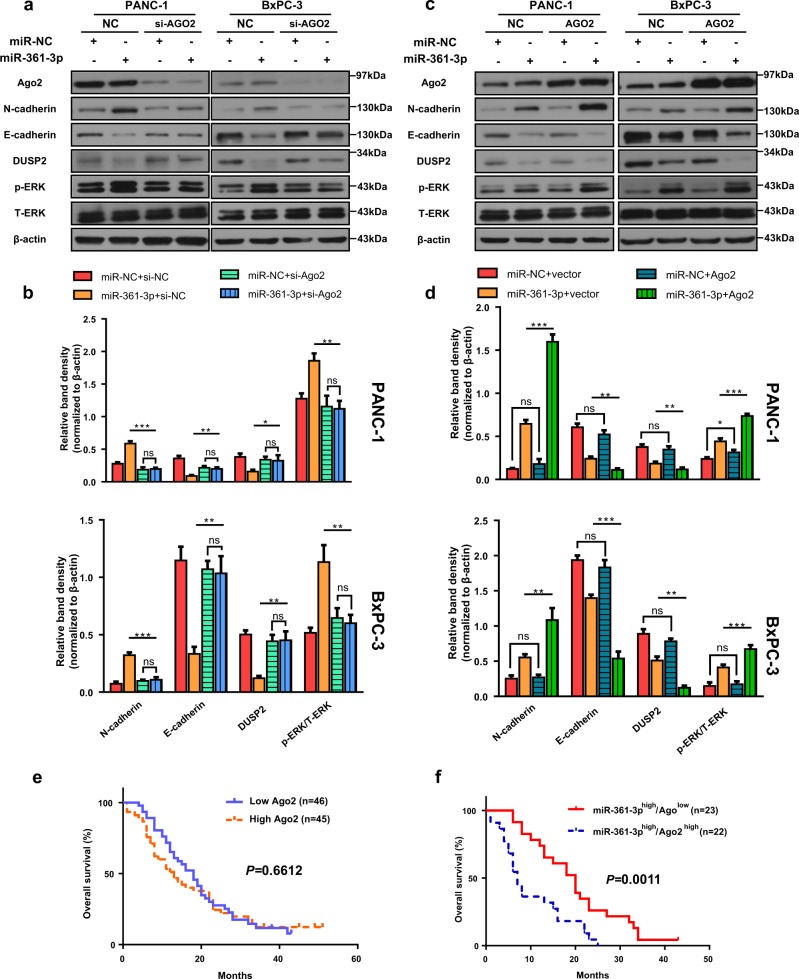
Ago2 enhanced miR-361-3p-induced EMT and ERK activation. **a**, **b** PANC-1 and BxPC-3 cells were transfected with miR-361-3p mimic, si-Ago2 alone, or the combination of miR-361-3p mimic and si-Ago2. Forty-eight hours after transfection, the cell lysates were subjected to western blot. **c**, **d** PANC-1 and BxPC-3 cells were transfected with miR-361-3p mimic, Ago2 plasmid alone, or the combination of miR-361-3p mimic and Ago2 plasmid. 48 h after transfection, the cell lysates were subjected to western blot. **e** Survival curve of high expression of Ago2 mRNA and low expression one (**f**) Survival curve of miR-361-3p^high^/Ago2^low^ group and miR-361-3p^high^/Ago2^high^ group. Data are shown as the mean ± SD of three replicates; *P < 0.05; **P < 0.01; ***P < 0.001; ns not significant

Argonaute 2 (Ago2) is the only mammalian Ago protein with endonucleolytic activity^24,34^, and it can mediate guiding target mRNA cleavage^35^. Moreover, Ago2 has been shown to regulate RNA interference^36^, miRNA processing^26^, microRNA abundance and stability^37^. Therefore, the effect of Ago2 in miR-361-3p-induced EMT progress was further tested. To determine whether Ago2 is required for miR-361-3p-mediated EMT and ERK1/2 activation, Ago2 was suppressed in miR-361-3p-overexpressing PANC-1 and BxPC-3 cells by siRNA. The miR-361-3p did not significantly affect the Ago2 expression (Fig. 5a, c) and failed to induce changes in phosphorylation of ERK1/2, E-cadherin, N-cadherin, and DUSP2 when Ago2 was knocked down (Fig. 5a, b). Since Ago1 is 80% identical to Ago2 but lacks a key catalytic residue^38^, we also evaluated the role of Ago1 in miR-361-3p-mediated knockdown. As shown in Fig. S6, Ago1, unlike Ago2, was not required for miR-361-3p-induced molecular functions. Thus, these results indicated that miR-361-3p regulates EMT and ERK1/2 activation in an Ago2-dependent manner.

Then, Ago2 was co-transfected with miR-361-3p mimic in PANC-1 and BxPC-3 cells. As shown in Fig. 5c, d, Ago2 alone did not affect or weakly affected the phosphorylation of ERK1/2, E-cadherin, N-cadherin, and DUSP2 compared to that in the negative control, but efficacy induced by miR-361-3p was strongly enhanced by Ago2 compared to that with miR-361-3p mimic alone. Given that the effect of Ago2 on miR-361-3p-mediated EMT described as above, we further analyzed the mRNA levels of Ago2 in PDAC tissues. No significant difference in overall survival was observed between Ago2^high^ and Ago2^low^ groups (Fig. 5e). Meanwhile, among the patients with high miR-361-3p levels, the group with Ago2^high^ displayed significantly poorer survival compared with that in the Ago2^low^ group (Fig. 5f). Collectively, we assumed that Ago2 could enhance the miR-361-3p-induced effect.

### Argonaute 2 enhanced miR-361-3p-mediated RNAi without regulating miR-361-3p biogenesis and stability

**Fig. 6:**
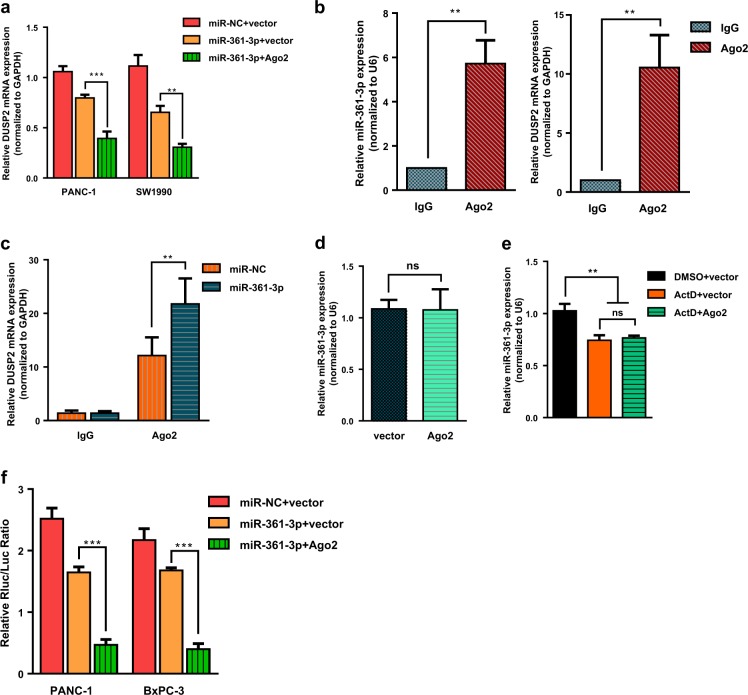
Argonaute 2 enhanced miR-361-3p-induced DUSP2 mRNA cleavage. **a** Relative expression of DUSP2 mRNA was assayed in PANC-1 and SW1990 cells transfected with miR-361-3p mimic or the combination of miR-361-3p mimic and Ago2 plasmid. **b** Relative expression of miR-361-3p (left) and DUSP2 mRNA (right) in the RNA of the nonspecific IgG or anti-Ago2 RIP. **c** Relative expression of DUSP2 mRNA in the RNA of the nonspecific IgG or anti-Ago2 RIP from cells transfected with miR-361-3p mimic. **d** Relative expression of miR-361-3p from cells transfected with Ago2 plasmid or vector. **e** Relative expression of miR-361-3p from cells after treatment with Actinomycin D alone or Actinomycin D plus Ago2 plasmid. **f** Luciferase activity assays was performed to demonstrate the binding efficiency of miR-361-3p and DUSP2 using luciferase reporter plasmids of pMIR-wild type-DUSP2 3′ UTR. Data are shown as the mean ± SD of three replicates; *P < 0.05; **P < 0.01; ***P < 0.001; ns not significant

Consistent with previous immunoblot results (Fig. 5a–d), DUSP2 mRNA levels were also notably decreased in the co-transfection group (Fig. 6a) compared with that in the miR-361-3p-alone group. To explore the mechanism that Ago2 enhanced miR-361-3p-mediated phenotype, RIP was performed to identify the relationship between Ago2 and miR-361-3p, and DUSP2 mRNA respectively. Analysis of the RIP products by qPCR showed that Ago2 can specifically interact with miR-361-3p and DUSP2 mRNA respectively (Fig. 6a). Furthermore, Ago2 showed increased association with the DUSP2 mRNA after miR-361-3p mimic transfection (Fig. 6b). Combined with the results above, we suggest that Ago2 can directly enhance miR-361-3p-induced DUSP2 mRNA degradation. However, Ago2 did not promote miR-361-3p biogenesis (Fig. 6d), and for further study, transcription inhibitor Actinomycin D (ActD) was used to test whether Ago2 mediates miR-361-3p stability. As shown in Fig. 6e, after treatment with ActD for 24 h, no significant enhanced stability of miR-361-3p was observed following Ago2 transfection. Hence, we deduced that Ago2 did not regulate miR-361-3p-induced DUSP2 mRNA decay through mediating miRNA biogenesis and stability.

Ago2 has also been shown to enhance miRNA-mediated knockdown with perfectly matched binding sites^36^. Because of perfect complementarity between the miR-361-3p and 3′ UTR of DUSP2 mRNA, the wild-type DUSP2 Luciferase construct was used to verify Ago2-enhanced knockdown. As expected, the reporter activity was suppressed by miR-361-3p, and tremendously suppressed by co-expression with Ago2 (Fig. 6f). Therefore, we considered that Ago2 might promote miR-361-3p-induced DUSP2 mRNA decay by enhancing its knockdown efficacy.

## Discussion

PDAC is associated with an extremely poor prognosis, for which mortality approximately parallels incidence in both the USA^1^ and China^39^. Early invasion and metastasis contributes to very few opportunities for radical excision and poor survival rates. Accumulating evidence reports that the EMT process plays a key role in a variety of types of tumors’ invasiveness and metastasis, including PDAC. However, the mechanisms beneath such malignant transition have not yet been completely demonstrated. Emerging reports about miRNA behavior revealed its indispensable roles in carcinogenesis and development. The present study provides clinical and experimental evidence to metastasis-promoting role of miR-361-3p in PDAC. We found that a high level of the miR-361-3p is associated with an advanced stage of tumor and a shorter overall survival. Moreover, in vitro experiments showed that miR-361-3p expression was positively correlated with metastasis. Consistently, the results that miR-361-3p knockdown inhibited the metastasis formation were demonstrated by two different in vivo models using lentiviral and antagomir. Thus, our results identified miR-361-3p as a potential oncomiR in promoting PDAC metastasis. Anti-tumor effects of miR-361-3p were also reported in non-small-cell lung cancer by targeting SH2B1^40^. To explain why miR-361-3p functions in a different way in PDAC, we investigated the baseline expression (Fig. S4b) and biological function of SH2B1 in PDAC and demonstrated that loss of SH2B1 inhibited migration and invasion (Fig. S4d), and reversed EMT in PDAC cells (Fig. S4e). However, unlike in previous articles, our data provide no evidence to support that miR-361-3p could regulate SH2B1 (Fig. S4a), at least in pancreatic cancer. Actually, diversiform or even opposite effects of one particular microRNA in different cancer is quite common^41^, especially when considering that one single miRNA might regulate tens to hundreds of different genes and that the biological phenotypes of such miRNA are influenced by the specific genetic backgrounds of different tumors. We also identified miR-361-3p as a novel EMT inducer in PDAC cells. In SW1990 and PANC-1 cells, ectopic expression of miR-361-3p led to EMT, whereas miR-361-3p knockdown yielded opposite effects.

In an effort to explore the mechanisms, our results showed that overexpression of miR-361-3p promoted activation of the ERK pathway, widely verified to be connected with EMT^10,15,42^. The ERK pathway is also required in TGFβ-1/H-Ras-induced Snail transcription factor^43^, recognized for involvement in EMT by directly binding the E-cadherin/CDH1 gene promoter^44^. Similarly, the present study identified that miR-361-3p-induced EMT was mediated via ERK activation. Herein, such results may suggest a general role of the ERK pathway in regulating the EMT process in PDAC.

After confirming the role of the ERK pathway in miR-361-3p-mediated EMT, we further demonstrated that DUSP2 was a direct target of miR-361-3p. Our data showed that miR-361-3p directly regulates DUSP2 expression by degrading DUSP2 mRNA by perfect complementarity between miR-361-3p and its 3′ UTR. DUSP2, originally named phosphatase of activated cells 1 (PAC-1), is a member of the dual-specificity protein phosphatase subfamily that negatively regulates MAPKs by dephosphorylating both the serine/threonine and tyrosine residues^45^. Moreover, downregulation of DUSP2 induced by hypoxia/HIF1α or promoter hypermethylation is associated with malignant tumor phenotype^45,46^. Consistent with our data, we not only demonstrated that restoration of DUSP2 abolishes miR-361-3p-induced EMT and ERK activation but also verified the direct interaction between DUSP2 and ERK1/2 in PDAC cells and tissues. Together, the above results comprehensively demonstrated that miR-361-3p regulates ERK-induced EMT by targeting DUSP2.

Ago2 protein, the only Ago protein with endonucleolytic activity, plays key roles in RNA interference and endogenous miRNAs biogenesis, which suggests coordinated regulation of microRNA expression and function^26^. The current study demonstrated that Ago2 is required in miR-361-3p-induced EMT, notably enhanced by Ago2 introduction. It was reported that Ago2 stabilized mature miRNA stability and increased miRNA half-life^37^. However, as shown in our study, Ago2 alone did not significantly enhance miR-361-3p abundance or stability, indicating that Ago2 regulation of miRNA stability or abundance is not a universal phenomenon, especially when poorly conserved. In addition to mediating miRNA abundance, Ago2 also dramatically enhanced RNAi efficacy towards mRNA targets with perfectly matched binding sites; such effect was independent of its regulation of miRNA expression^36^. As 7 perfectly matched base pairs were seen in the seed sequence, we speculated whether Ago2 might regulate miR-361-3p-induced gene knockdown via the same mechanism. Consistent with previous findings, our results showed that Ago2 enhanced miR-361-3p-inference potency without regulating miR-361-3p stability or biogenesis.

**Fig. 7:**
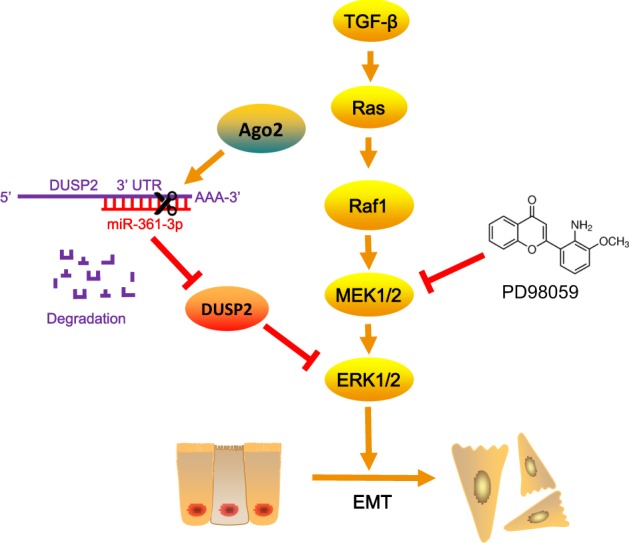
**Proposed model of miR-361-3p/DUSP2/ERK axis in EMT process in PDAC**

On the basis of previous studies of miR-361-3p, we provided clinical and experimental evidence of the oncomiR role of miR-361-3p and revealed a novel miR-361-3p/DUSP2/ERK axis in regulating PDAC EMT in vivo and in vitro completely. In addition, we demonstrated that co-expression of Ago2 enhanced miR-361-3p-induced EMT and DUSP2 mRNA degradation (Fig. 7); miR-361-3p plus Ago2 may serve as a better prognostic biomarker useful for prediction of prognosis in patients with PDAC.

## Materials and methods

### Cell lines and reagents

The human pancreatic cancer cell lines BxPC-3 and PANC-1 and human pancreatic duct epithelial cells (HPDE6-C7) were purchased from the American type culture collection, and CFPAC-1 and SW1990 were obtained from the Type Culture Collection of the Chinese Academy of Sciences (Shanghai, China). BxPC-3 and PANC-1 cell lines were routinely cultured in RPMI 1640 medium (HyClone, USA), and CFPAC-1, SW1990 and HPDE6-C7 cell lines was grown in Dulbecco’s modified Eagle’s medium (Gibco, USA) supplemented with 10% fetal bovine serum (Gibco, USA), penicillin (100 U/ml) and streptomycin (100 mg/ml). All cells were cultured at 37 °C with 5% CO_2_.

The following reagents were used: protease inhibitor cocktail (Sigma-Aldrich, USA); phosphatase inhibitor (Roche, Shanghai, China); RNase OUT (Thermo Fisher Scientific, Waltham, MA); MAPKK inhibitor PD 98059 (Sigma-Aldrich, St. Louis, MO); Proteinase K (Sigma-Aldrich); and Actinomycin D (Sigma-Aldrich).

### Patients and specimens

PDAC and normal pancreatic tissue were obtained from 65 patients who underwent pancreatic resection in the Department of Pancreatic and Biliary Surgery (The First Affiliated Hospital of Harbin Medical University, Harbin, Heilongjiang, China) from January 2008 to January 2014. Samples were frozen in liquid nitrogen immediately after surgical resection for further RNA extraction. The patients’ clinical characteristics are shown in Supplementary Table S1. This study was approved by the Ethics Committee of the First Affiliated Hospital of Harbin Medical University.

### Transfection

MiR-361-3p mimic (miR-361-3p), mimic negative control (miR-NC), miR-361-3p-specific inhibitor (miR-361-3p-in), miRNA inhibitor control (miR-in NC), a miR-361-3p antagomir (miR-361-3p-anta), antagomir negative control, siRNA for DUSP2 (si-DUSP2), AGO2 (si-AGO2), AGO1 (si-AGO1), SH2B1 (si-SH2B1), and siRNA negative control (si-NC) were purchased from RIBOBIO (Guangzhou, China). To induce overexpression of DUSP2 and AGO2, human DUSP2 (NM_004418) and AGO2 (NM_012154) cDNA were cloned into the GV230 plasmid (Genechem, Shanghai, China)

For transient transfection, the cells were seeded in six-well plates, and 50 nM mimic/miRNA-inhibitor/siRNA or 2 μg plasmids were transfected into the cells using Lipofectamine 2000 (Thermo Fisher Scientific). BxPC-3-Luc cells were infected with lentiviral-expressing GV280 plasmid containing miR-361-3p-specific shRNA (Lv-sh-miR-361-3p) or the control shRNA (Lv-sh-NC) (Genechem, Shanghai, China). Stable clones were selected via puromycin for 2 weeks. The efficiency of all transfection was evaluated by qRT-PCR and/or western blot. The target sequences of siRNAs are listed in Supplementary Table S2.

### Wound healing assay

The wound healing assay was performed as described previously^47^. Briefly, transfected cells grown in six-well plates were pretreated with mitomycin C (10 μg/mL) 2 h before an artificial “wound” created with a 200 μL pipette tip at 0 h and then incubated in serum-free medium (for PANC-1, SW1990 and CFPAC-1) or 1% serum medium (for BxPC-3). Images were acquired at 0 h and 24 h at 10× on Olympus microscope. The percentage of wound closure was estimated by ImageJ software.

### Transwell assay

The Transwell assays were performed as described previously^47^. The motility and invasiveness of cells exposed to DAC were assayed using 8 μm pore size Falcon^®^ inserts coated with or without Matrigel (BD Biosciences, USA). Cells suspended in serum-free medium were seeded in the upper part of the Transwell unit and allowed to invade for 24 h. The lower part of the Transwell unit was filled with 500 μL medium containing 10% FBS. After incubation, non-invasive cells on the upper part of the membrane were removed with a cotton swab. Invasive cells on the bottom surface of the membrane were fixed in methanol and then stained with crystal violet. The number of cells in five randomly selected fields (20×) was counted, and all assays were performed in triplicate.

### Colony formation assay

The colony formation assay was performed as described previously^47^. Five-hundred cells transfected with Lv-sh-miR-361-3p and negative control lentiviral vectors were cultured in 6-well plates. The medium was changed every 3 days. After day 14, the colonies were counted after fixation in methanol for 10 min with 1% crystal violet staining. The colonies were counted manually in five fields (10×, Olympus Co., Tokyo, Japan).

### Cell viability assay

Cell viability was monitored using Cell Counting Kit-8 (CCK-8) (Dojindo Laboratories) as described previously^48^. In brief, after transfection, cells were treated with a gradient concentration of gemcitabine (GEMZAR, Eli Lilly and Company) for 72 h. Then, after incubation with CCK-8 for 1.5 h, the absorbance was measured at 450 nm (ELx808, BioTek, USA); IC50 values referring to the gemcitabine concentration were calculated using GraphPad Prism 6.01 software.

### EdU proliferation assay

Cell proliferation was also determined by a 5-ethynyl-2′-deoxyuridine (EdU) assay (RIBOBIO, Guangzhou, China). The EdU proliferation assay was performed as described previously^47^. Following transfection, 5 × 10^3^ infected cells from two groups were seeded in 96-well plates, and then, the cells were treated following the manufacturer’s instructions. The cells were visualized with a fluorescence microscope (10×, Olympus Co., Tokyo, Japan).

### Luciferase reporter assay

To directly detect the regulation of DUSP2 by miR-361-3p, the full-length 677 bp 3′ UTR of wild-DUSP2 (WT) and same length mutant-DUSP2 (MUT) were amplified and then cloned into pmiR-RB-Report^TM^ vector (RIBOBIO, Guangzhou, China). For the luciferase reporter assay, PANC-1 cells were co-transfected with 50 nM miR-361-3p mimic and 500 ng of Luciferase constructs, and BxPC-3 cells were transfected with 100 nM miR-361-3p inhibitor and 500 ng of Luciferase constructs according to the manufacturer’s protocol. The cells were harvested 24 h after transfection, and the luciferase activity with or without AGO2 overexpression was measured with a Dual-Luciferase Reporter Assay System (Promega, Madison, WI, USA), through Varioskan Flash Spectral Scanning Multimode Reader (Thermo Fisher Scientific). Firefly luciferase activity was used to normalize the transfection efficiency.

### RNA extraction and quantitative real-time PCR analyses

Total RNA was extracted and isolated from cell lines and frozen tumor specimens using AxyPrep Multisource Total RNA Miniprep Kit (Axygen Biosciences, USA) according to the manufacturer’s instructions and the first-strand cDNA was synthesized using the ReverTraAce qPCR RT Kit (FSQ-101, Toyobo Co. Ltd.) according to the manufacturer’s instructions. Quantitative real-time polymerase chain reaction (qRT-PCR) was performed as previously described^47^. Briefly, qRT-PCR (SYBR Green Assay, Roche Diagnostics GmbH) was performed on a 7500 FAST Real-Time PCR System (Applied Biosystems). The relative expression levels of miR-361-3p and mRNAs were calculated and quantified using the 2^−ΔΔT^ method after normalization for the expression of the control. U6 and GAPDH served as the endogenous controls, respectively. The primer sequences are described in Supplementary Table S3 and were purchased from Thermo Fisher Scientific and RIBOBIO.

### Western blotting

The methodology has been described previously^47,49^. In brief, total proteins from pancreatic cancer cells or tissues were extracted using RIPA buffer (Beyotime Institute of Biotechnology, Beijing, China) that contained protease inhibitor cocktail and phosphatase inhibitor and homogenized. Protein samples (20–40 μg/sample) were separated using SDS-PAGE and transferred onto polyvinylidene difluoride (PVDF) membranes. The membranes were blocked with 5% non-fat milk prior to incubation with primary antibodies, and subsequently with HRP-conjugated secondary antibodies (Santa Cruz Biotech, CA, USA). Protein bands were visualized with the enhanced chemiluminescence kits (Pierce Chemical, Rockford, IL, USA). β-actin was the loading control. Additionally, the level of protein expression was calibrated to the band density of β-actin.

### Co-immunoprecipitation (co-IP)

First, 2 × 10^7^ cells were lysed with 500 μL of ice-cold polysome lysis buffer (100 mM KCl, 4 mM MgCl_2_, 10 mM Hepes, pH 7.0, 0.5% Nonidet P-40, 1 mM DTT, 100 units/ml RNase OUT, 40 μL/mL complete protease inhibitor cocktail) for 10 min on ice. Then, the cell lysates were collected after centrifugation. DUSP2 antibodies (sc-32776, Santa Cruz, USA) or control immunoglobulin (IgG) (Santa Cruz, USA) were added to Protein G Agarose beads (EMD Millipore Corporation, CA, USA) and allowed to bind while rotating at 4 °C overnight (for DUSP2) or 2 h (for IgG). The lysates were pre-cleared with IgG antibody and then incubated with pre-coated beads for 2 h at RT on a rotator. The lysates were then spun down, and the pellet was saved for western blotting as described above.

### RNA-binding protein immunoprecipitation (RIP)

RNA-binding protein immunoprecipitation was carried out as previously described^47^, with some modifications. Cell lysates and beads pre-coated with Ago2 antibodies were prepared as described in the co-IP section. The complexes were incubated with 0.1% SDS and 0.5 mg/mL Proteinase K at 55 °C for 15 min. The supernatant was collected for RNA isolation using Trizol Ls Reagent (Thermo Scientific). Finally, qRT-PCR analyses of the RNA isolated from the immunoprecipitation (IP) material were further assessed as described above.

### Orthotopic and metastatic mice models

All animal studies were conducted under a protocol approved by the Institutional Review Board of the First Affiliated Hospital of Harbin Medical University. Female 6-week-old BALB/c nude mice were purchased from the Beijing Vital River Laboratory Animal Technology and maintained in SPF environment.

### Orthotopic pancreatic cancer model

Orthotopic tumor models were created as previously described^47^. Briefly, 5 × 10^6^ Bxpc-3-Luc cells transfected with Lv-miR-361-3p inhibitor (Lv-sh-miR-361-3p) or the negative control vector (Lv-NC) was injected into the right flank of nude mice. Then, 1 mm^3^ pieces of tumor harvested from two mice were translocated into two groups of mice’s pancreatic tails respectively. The animals were imaged weekly using the NightOWL II LB983 In vivo imaging system (BERTHOLD TECHNOLOGIES GmbH & Co. KG, Germany). Another two groups were orthotopically implanted with tumor pieces derived from normal BxPC-3 cells, followed by a succession of either PBS or 50 nmol antagomir-361-3p treatments via the tail vein injection, twice per week for 3 weeks. The numbers of visible metastatic lesions proved by pathology diagnosis afterwards in the gut, mesentery, liver, spleen and kidneys were recorded 4 weeks after xenograft procedures. The primary and metastatic pancreatic tumors were excised, weighed, and fixed in 4% paraformaldehyde.

### Liver metastasis model

Nude mice were anaesthetized with 1% pentobarbital, and left subcostal incisions were made to expose their spleens. A total of 5 × 10^6^ cells in 200 mL PBS were injected gently into spleen parenchyma, and then, the spleen was removed to avoid bleeding and intrasplenic tumor. After 5 weeks of implantation, the mice were euthanized and their livers were removed for counting metastatic nodules.

### In situ hybridization (ISH) analysis

Human PDAC tissues were fixed in 4% paraformaldehyde (pH 7.0–7.6, containing 0.1% DEPC) and then embedded in paraffin. A digoxigenin-labeled miR-361-3p oligonucleotide probe (5′-AAATCAGAATCACACCTGGGGGA-3′) and an ISH kit (MK10503) were obtained from Boster Biological Technology (Wuhan, China). Slides were deparaffinized and incubated for 20 min at room temperature with Pepsin; subsequently, the sections were prehybridized in a humid chamber at 38–42 °C for 2 h. Then, the tissues were hybridized overnight with a miR-361-3p probe at 38-42 °C. After hybridization, the slides were washed with graded-diluted sodium citrate buffer (SSC) at 37 °C for 40 min, followed by incubation with an antibody against digoxigenin at 37 °C for 60 min. These sections were then incubated with SABC-POD kit (Boster, Wuhan, China), and hybridization signals were visualized using diaminobenzidine (DAB) (Boster, Wuhan, China). Finally, the tissues were counterstained with hematoxylin, mounted and imaged.

### Immunohistochemistry (IHC) staining

The immunohistochemical staining protocol has been described previously^47,50,51^. In brief, paraffin-embedded tissue sections (5 μm) were immunostained with anti-DUSP2 (sc-32776, Santa Cruz), anti-E-cadherin (3195, Cell Signaling Technology), anti-N-cadherin (610920, BD Biosciences), anti-Vimentin (10366-1-AP, Proteintech), and anti-Phospho-ERK1/2 (4370, Cell Signaling Technology). The number of positive cells was counted in five randomly selected microscopic fields (×10, Nikon, Japan).

### Statistical analysis

Statistical analysis was performed with SPSS 19.0 software or GraphPad Prism 6.01 software. The data are shown as the mean ± standard deviation (SD). Pearson analysis, Kaplan–Meier survival analysis, one-way ANOVA and Student *t*-test were used to evaluate statistical significance. Differences are considered statistically significant when *P* < 0.05.

## Electronic supplementary material

Table S1. Clinical correlation between miR-361-3p expression and clinical and pathological characteristics in PDAC patients

Table S2. The target sequences of siRNAs used in transfection

Table S3. Sequences of primers used in Real-time RT-PCR

Figure S1. Expression of miR-361-3p in cell lines, tumors from orthotopic mouse models

Figure S2. MiR-361-3p does not have a significant effect on PDAC proliferation

Figure S3. MiR-361-3p knock-down reversed EMT and ERK activation in vivo and in vivo, and miR-361-3p enhanced resistance to gemcitabine treatment

Figure S4. SH2B1 silencing inhibited EMT and MiR-361-3p had no effect on SH2B1 expression

Figure S5. MiR-361-3p knock-down restored DUSP2

Figure S6. Ago1 was not required in miR-361-3p-mediated functions

Supplementary Figure Legend
