# Supplementary material for: MiR-361-3p regulates ERK1/2-induced EMT via DUSP2 mRNA degradation in pancreatic ductal adenocarcinoma
Source: Cell Death Dis. 2018 Jul 24;9(8):807. doi: 10.1038/s41419-018-0839-8 (PMC6057920; doi:10.1038/s41419-018-0839-8)
Supplement: Supplementary file 1 — Table S1. Clinical correlation between miR-361-3p expression and clinical and pathological characteristics in PDAC patients [file 41419_2018_839_MOESM1_ESM.docx]

**Table S1. Clinical correlation between miR-361-3p expression and clinical and pathological characteristics in PDAC patients.**

| **Clinical characteristics** | **Total** | **miR-361-3p expression** | | ***P* value** |
| --- | --- | --- | --- | --- |
|  |  | **Low** | **High** |  |
| Age (years) |  |  |  |  |
| ＜60 | 53 | 27 | 26 | 1.000 |
| ≥60 | 38 | 19 | 19 |  |
| Gender |  |  |  |  |
| Male | 68 | 33 | 35 | 0.6308 |
| Female | 23 | 13 | 10 |  |
| TNM stage |  |  |  |  |
| I+IIa | 63 | 37 | 26 | 0.0240 |
| IIb+III | 28 | 9 | 19 |  |
| Nodal metastasis |  |  |  | 0.0404 |
| Yes | 22 | 9 | 18 |  |
| No | 43 | 37 | 27 |  |
