# Supplementary material for: MiR-361-3p regulates ERK1/2-induced EMT via DUSP2 mRNA degradation in pancreatic ductal adenocarcinoma
Source: Cell Death Dis. 2018 Jul 24;9(8):807. doi: 10.1038/s41419-018-0839-8 (PMC6057920; doi:10.1038/s41419-018-0839-8)
Supplement: Supplementary file 2 — Table S2. The target sequences of siRNAs used in transfection [file 41419_2018_839_MOESM2_ESM.docx]

**Table S2. The target sequences of siRNAs used in transfection**

| Product Number | Product Name | Target Sequence |
| --- | --- | --- |
| siN05815122147-1-5 | siR-Ribo™  Negative Control | N/A |
| siB08722110729 | si-human-DUSP2 | CCACCATCTGTCTGGCATA |
| siB0837163230 | si-human-Ago2 | GCAGGACAAAGATGTATTA |
| siG150821104921 | si-h-SH2B1_001 | GAAGGTCCATCCGAGTATA |
| siG150821104936 | si-h-SH2B1_002 | GAAGTCGCCTGGAGTTCTT |
| siG150821104949 | si-h-SH2B1_003 | GGGACCTCATTCCTTACAA |
| stB0001666A | si-h-AGO1_001 | TCCCTAAGATCGACGTGTA |
| stB0001666B | si-h-AGO1_002 | AGCCGACCATCCCATTACT |
| stB0001666C | si-h-AGO1_003 | GCTGTGCCACACTTACGTA |
