# Supplementary material for: MiR-361-3p regulates ERK1/2-induced EMT via DUSP2 mRNA degradation in pancreatic ductal adenocarcinoma
Source: Cell Death Dis. 2018 Jul 24;9(8):807. doi: 10.1038/s41419-018-0839-8 (PMC6057920; doi:10.1038/s41419-018-0839-8)
Supplement: Supplementary file 3 — Table S3. Sequences of primers used in Real-time RT-PCR [file 41419_2018_839_MOESM3_ESM.docx]

**Table S3. Sequences of primers used in Real-time RT-PCR**

| Primer Name: | Sequences (5’ to 3’) |
| --- | --- |
| hsa-GAPDH-Forward | CCTCTGACTTCAACAGCGACAC |
| hsa-GAPDH-Reverse | TGGTCCAGGGGTCTTACTCC |
| hsa-DUSP2-Forward | TACTTCCTGCGAGGAGGCTT |
| hsa-DUSP2-Reverse | TAGACAGGAGCCCTGGAGTC |
| hsa-Ago2-Forward | CCCGCATCATCTTCTACCGC |
| hsa-Ago2-Reverse | GCTTGTCCCCCGCTCGTT |
