# Supplementary figures and images for: MiR-361-3p regulates ERK1/2-induced EMT via DUSP2 mRNA degradation in pancreatic ductal adenocarcinoma
Source: Cell Death Dis. 2018 Jul 24;9(8):807. doi: 10.1038/s41419-018-0839-8 (PMC6057920; doi:10.1038/s41419-018-0839-8)

**a**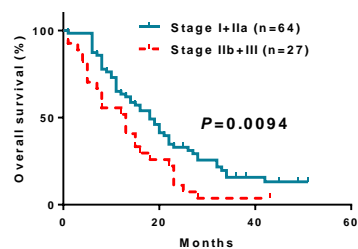**b**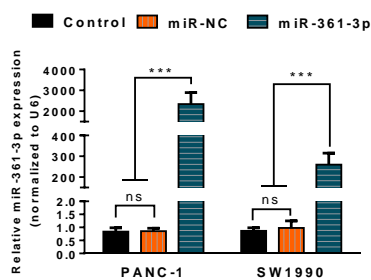**c**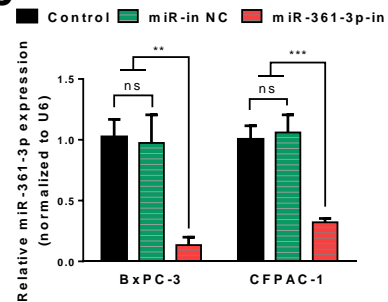**d**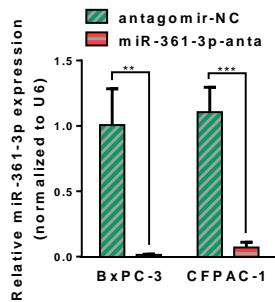**e**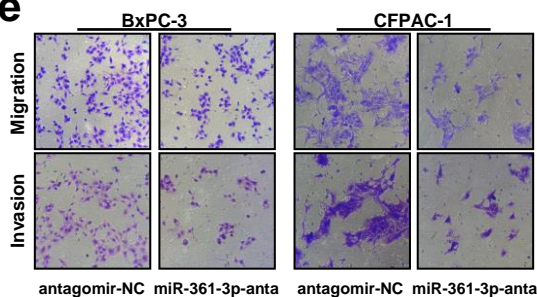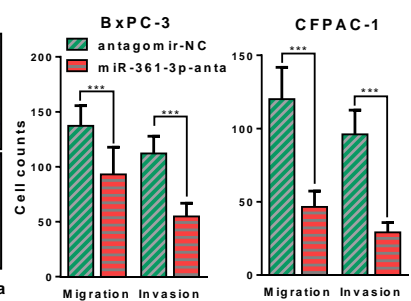**f**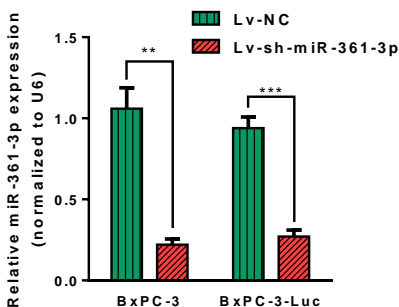**g**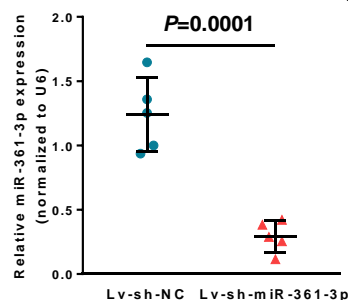**h**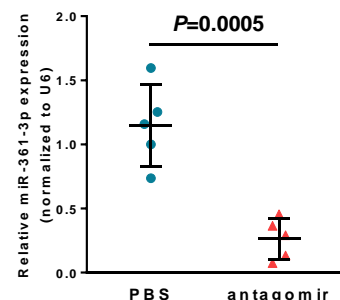**i**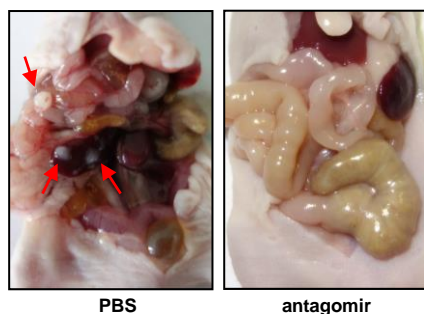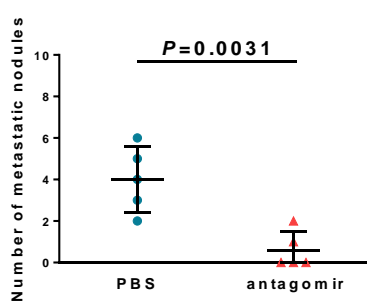

Supplement: Supplementary file 4 — Figure S1. Expression of miR-361-3p in cell lines, tumors from orthotopic mouse models [file 41419_2018_839_MOESM4_ESM.pdf]

**a**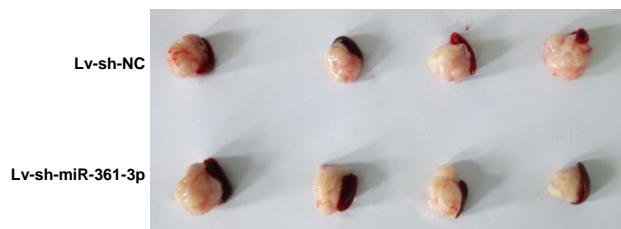**b**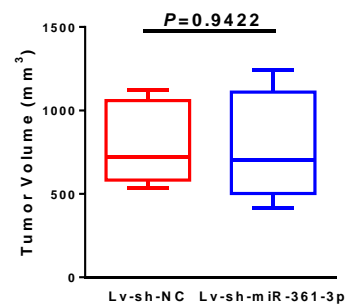**c**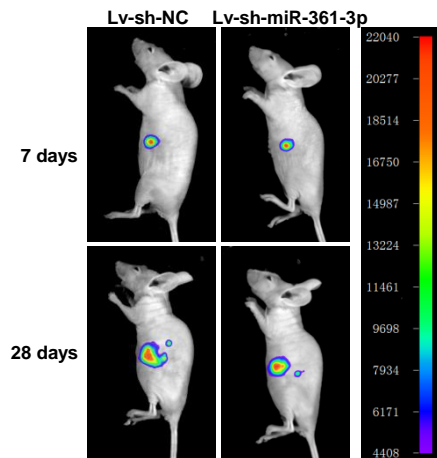**d**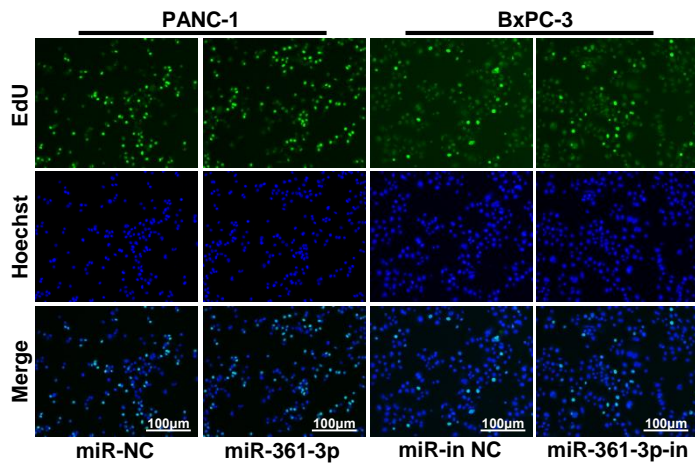**e**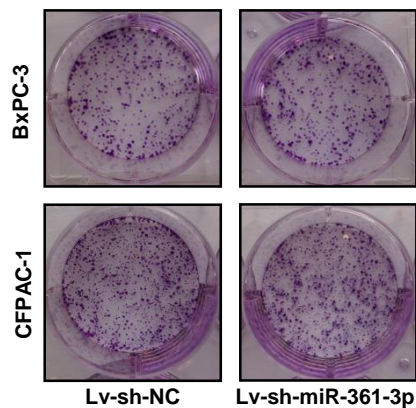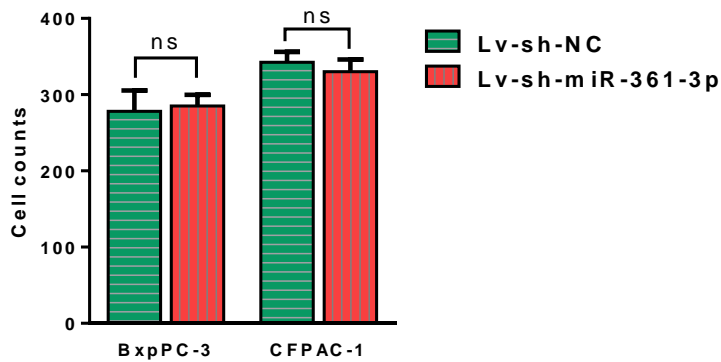**f**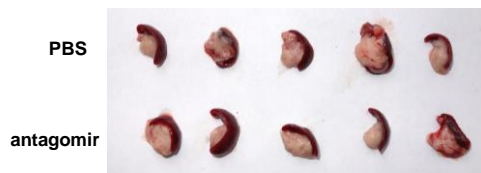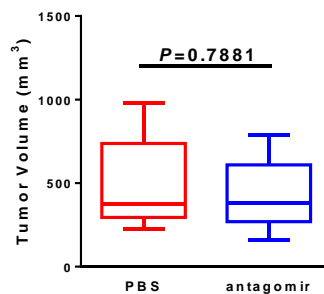

Supplement: Supplementary file 5 — Figure S2. MiR-361-3p does not have a significant effect on PDAC proliferation [file 41419_2018_839_MOESM5_ESM.pdf]

**a**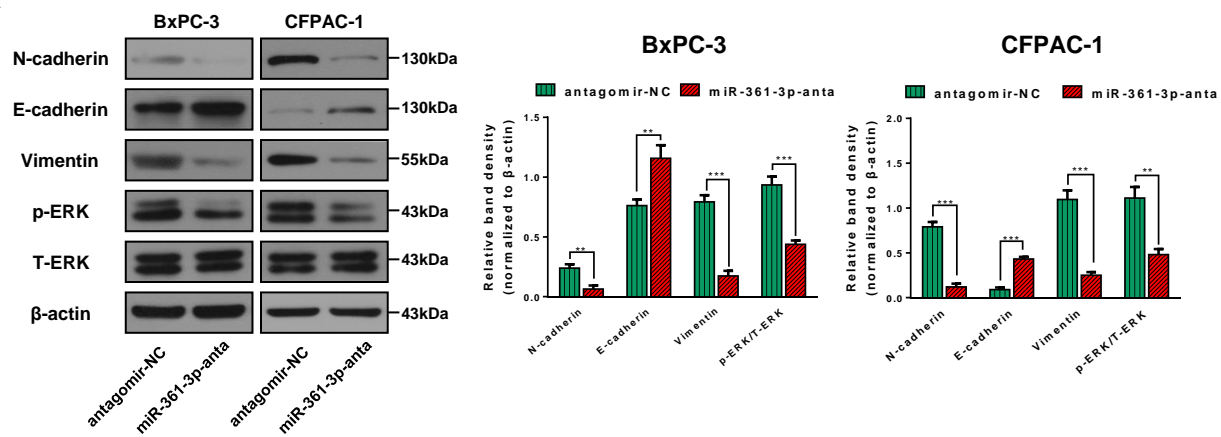**b**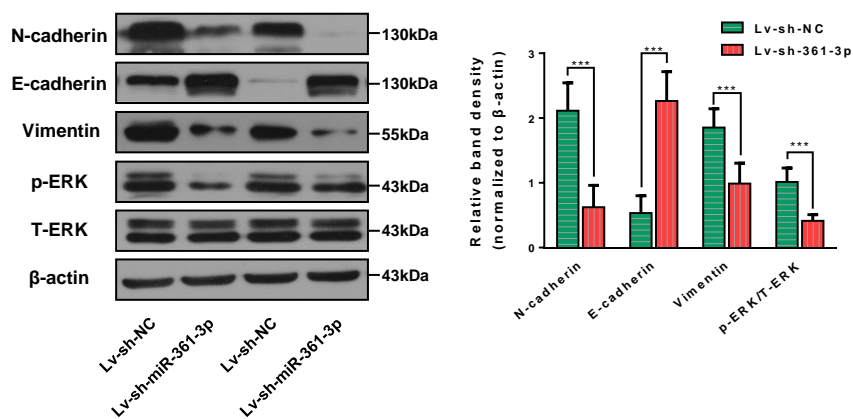**c**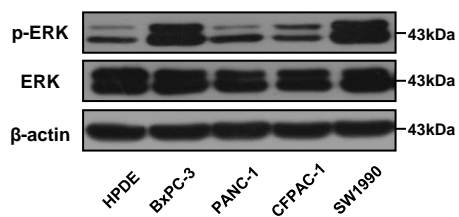**d**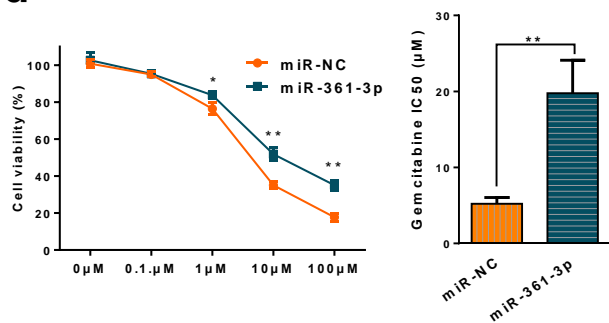**e**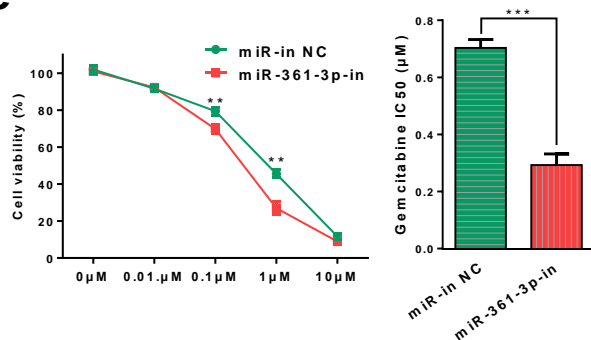

Supplement: Supplementary file 6 — Figure S3. MiR-361-3p knock-down reversed EMT and ERK activation in vivo and in vivo, and miR-361-3p enhanced resistance to gemcitabine treatment [file 41419_2018_839_MOESM6_ESM.pdf]

**a**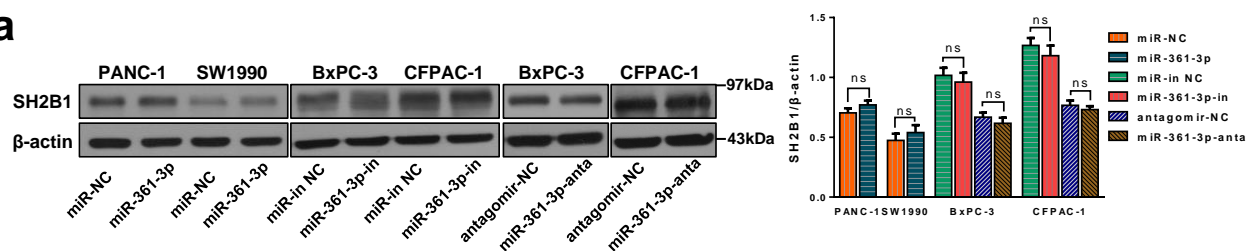**b**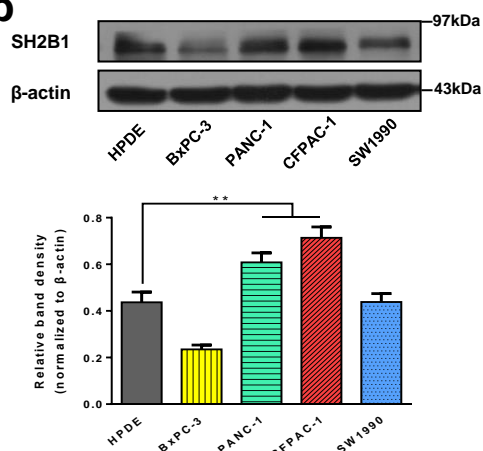**c**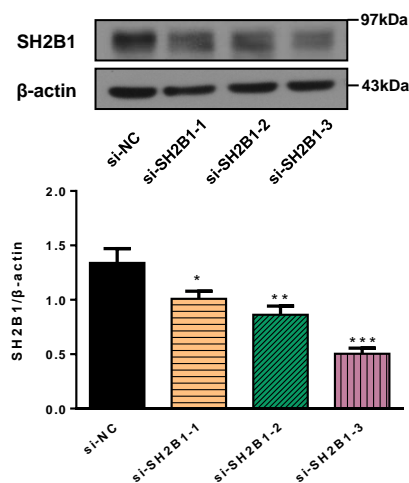**d**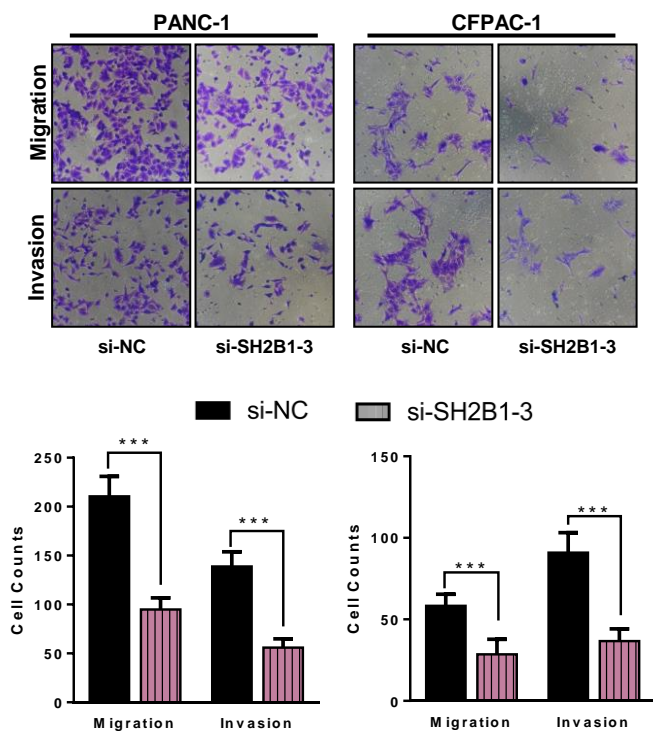**e**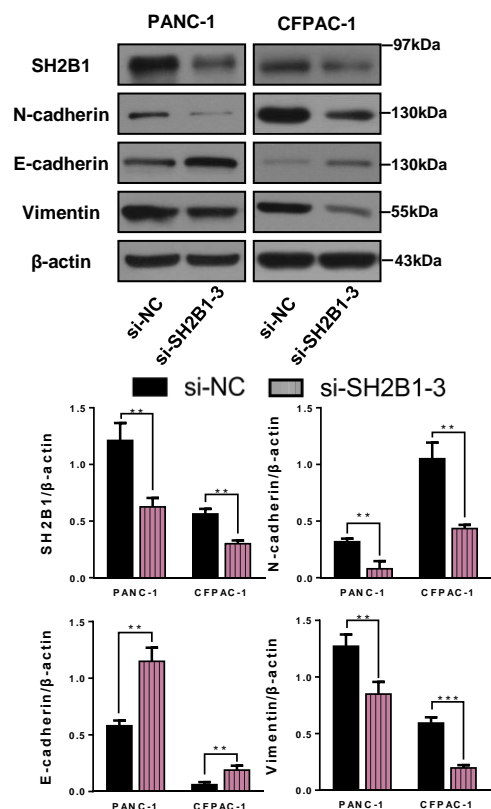

Supplement: Supplementary file 7 — Figure S4. SH2B1 silencing inhibited EMT and MiR-361-3p had no effect on SH2B1 expression [file 41419_2018_839_MOESM7_ESM.pdf]

**a**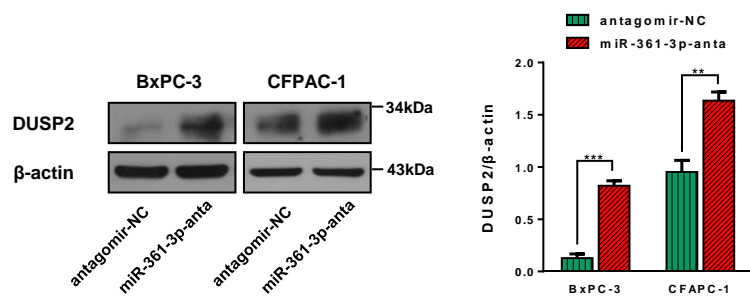**b**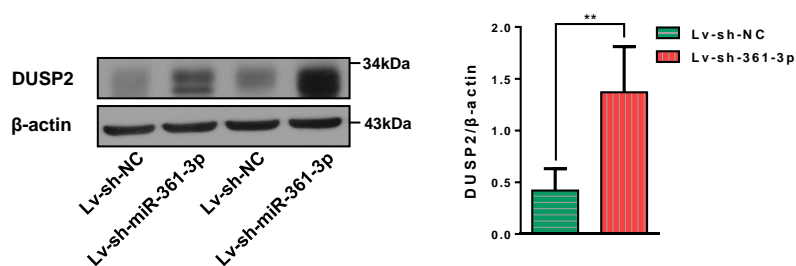

Supplement: Supplementary file 8 — Figure S5. MiR-361-3p knock-down restored DUSP2 [file 41419_2018_839_MOESM8_ESM.pdf]

**a**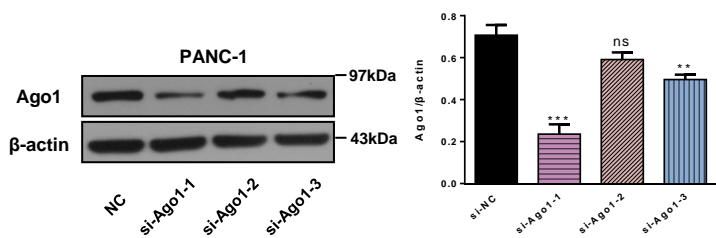**b**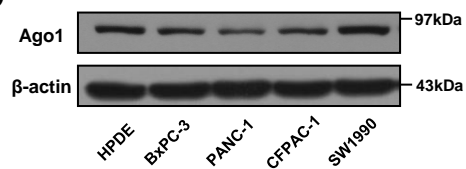**c**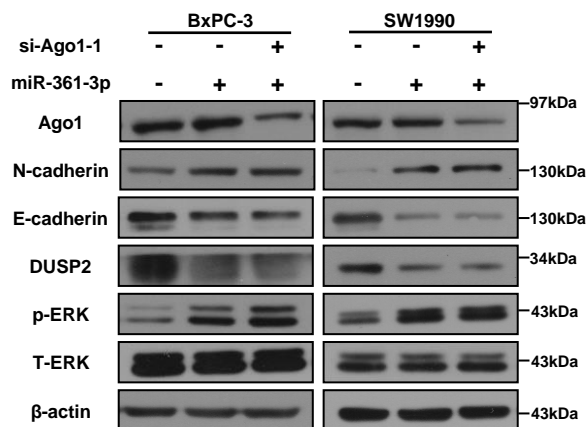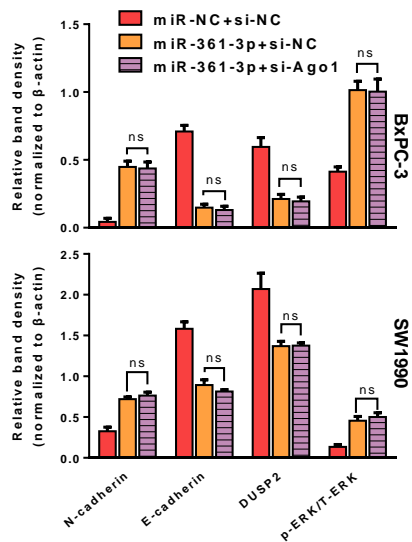

Supplement: Supplementary file 9 — Figure S6. Ago1 was not required in miR-361-3p-mediated functions [file 41419_2018_839_MOESM9_ESM.pdf]
