# Supplementary material for: MiR-361-3p regulates ERK1/2-induced EMT via DUSP2 mRNA degradation in pancreatic ductal adenocarcinoma
Source: Cell Death Dis. 2018 Jul 24;9(8):807. doi: 10.1038/s41419-018-0839-8 (PMC6057920; doi:10.1038/s41419-018-0839-8)
Supplement: Supplementary file 10 — Supplementary Figure Legend [file 41419_2018_839_MOESM10_ESM.docx]

**Supplementary Figure S1. Expression of miR-361-3p in cell lines, tumors from orthotopic mouse models.**

**a** Kaplan-Meier analyses of postoperative survival in PDAC patients with I / IIa and IIb / III stage in 91 PDAC tissues. **b-d** Relative miR-361-3p expression measured by qRT-PCR after treatment with miR-361-3p mimic, miR-361-3p inhibitor or miR-361-3p antagomir. **e** Transwell assay was performed to validate the of phenotype of miR-361-3p by using miR-361-3p antagomir. **f** Expression levels of miR-361-3p in stable cell lines. **g-h** Expression levels of miR-361-3p in tumors from orthotopic mouse models. **i** Representative images of orthotopic xenograft pancreatic cancer mouse models from PBS and miR-361-3p antagomir injection group (left panel) and metastatic nodes were calculated (right panel), red arrows indicate metastatic lesions. The statistical significance between different groups was calculated with Student *t* test. Data are shown as the mean ± SD of three replicates; **, *P* < 0.01; ***, *P* < 0.001; ns, not significant.

.

**Supplementary Figure S2. MiR-361-3p does not have a significant effect on PDAC proliferation.**

**a-b, f** At day 28, all mice were sacrificed and the primary tumors were removed and the tumor volume was evaluated. **c** Representative bioluminescence imaging of orthotopic xenograft mice at the day of 7 and 28. **d-e** the proliferative capacity of pancreatic cancer cells was determined by EdU assays (10×) and colony formation assay. Data are shown as the mean ± SD of three replicates; *, *P* < 0.05; **, *P* <0.01; ***, *P* < 0.001; ns, not significant.

**Supplementary Figure S3. MiR-361-3p knock-down reversed EMT and ERK activation *in vivo* and *in vivo,* and miR-361-3p enhanced resistance to gemcitabine treatment.**

**a-b** The expression of Vimentin, N-cadherin, E-cadherin, phospho-ERK1/2 and total ERK1/2 was detected by Western blot assays in (a) BxPC-3 and CFPAC-1 transiently transfected with miR-361-3p antagomir and in (b) tumor xenografts from the miR-361b-3p knock-down and negative control groups. **c** Baseline expression of p-ERK and total ERK was shown in Bxpc-3, Panc-1, CFPAC, SW1990, and HPDE cell lines. **d-e** CCK-8 assay and the corresponding IC50 of gemcitabine in pancreatic cancer cells transfected with mimic or miRNA-inhibitor. Data are shown as the mean ± SD of three replicates; **, *P* <0.01; ***, *P* < 0.001.

**Supplementary Figure S4. SH2B1 silencing inhibited EMT and MiR-361-3p had no effect on SH2B1 expression.**

**a** Relative expression of SH2B1 protein after treatment with miR-361-3p mimic, inhibitor and antagomir. **b** Baseline expression of SH2B1 was shown in HPDE, Bxpc-3, Panc-1, CFPAC, and SW1990cell lines. **c** Expression levels of SH2B1 after treatment with three different siRNAs. **d** Transwell assay was performed to test the effect of SH2B1 on PDAC cells migration and invasion *in vitro*. e The expression of Vimentin, N-cadherin and E-cadherin was detected by Western blot assays in PANC-1 and CFPAC-1 transfected with SH2B1 siRNA. Data are shown as the mean ± SD of three replicates; *, *P* < 0.05; **, *P* <0.01; ***, *P* < 0.001; ns, not significant.

**Supplementary Figure S5. MiR-361-3p knock-down restored DUSP2**

**a-b** The expression of DUSP2 was detected by Western blot assays in (a) BxPC-3 and CFPAC-1 transiently transfected with miR-361-3p antagomir and in (b) tumor xenografts from the miR-361b-3p knock-down and negative control groups. Data are shown as the mean ± SD of three replicates; **, *P* <0.01; ***, *P* < 0.001.

**Supplementary Figure S6. Ago1 was not required in miR-361-3p-mediated functions.**

**a** Expression levels of Ago1 after treatment with three different siRNAs. **b** Baseline expression of SH2B1 was shown in HPDE, Bxpc-3, Panc-1, CFPAC, and SW1990cell lines. **c** SW1990 and BxPC-3 cells were transfected with miR-361-3p mimic or the combination of miR-361-3p mimic and si-Ago1 48 h after transfection, the cell lysates were subjected to western blot. Data are shown as the mean ± SD of three replicates; **, *P* <0.01; ***, *P* < 0.001; ns, not significant.
